# Adaptability and learning Intraprofessional collaboration of residents during the COVID-19 pandemic

**DOI:** 10.1186/s12909-022-03868-9

**Published:** 2022-11-12

**Authors:** C. L. Bus, R. van der Gulden, M. Bolk, J. de Graaf, M. van den Hurk, N. N. D. Scherpbier-de Haan, C. R. M. G. Fluit, W. Kuijer-Siebelink, N. Looman

**Affiliations:** 1 Radboudumc Health Academy, Department of Research on Learning and Education, Radboud University Medical Centre, Nijmegen, the Netherlands; 2 School of Education, Department of Research on responsive vocational and professional education, HAN University of Applied Sciences, Nijmegen, the Netherlands; 3 Radboud Institute for Health Sciences, Department of Primary and Community Care, Radboud University Medical Center, Nijmegen, the Netherlands; 4 Dutch Association of Medical Specialists, Utrecht, the Netherlands; 5 Radboudumc Health Academy, Division of Post Graduate Medical Education, Radboud University Medical Centre, Nijmegen, the Netherlands; 6 Department of Educational Sciences, Faculty of Social Sciences, Radboud University, Nijmegen, the Netherlands; 7 Department of General Practice and Elderly Care Medicine, University Medical Centre Groningen, Groningen, the Netherlands

**Keywords:** Adaptability, Collective uncertainty, Crisis, (learning) intraprofessional collaboration, Pandemic, Postgraduate training, Resident, Social cohesion, Supervision

## Abstract

**Context:**

The COVID-19 pandemic created a worldwide public health emergency, in which hospitals created new COVID departments and doctors from different disciplines had to work together. In the Netherlands, a large proportion of doctors in these departments were residents. With knowledge of the disease developing only gradually, the influx of COVID-19 patients called for adaptability, innovative work behavior, and intraprofessional collaboration (intraPC) between residents and between residents and medical specialists.

**Research goal:**

This study investigates how the delivery of COVID-19 care in hospital settings altered the way residents develop their sense of adaptability and intraPC during their training.

**Methods:**

Sixteen semi-structured interviews were conducted with residents and medical specialists from various disciplines who worked at a COVID department or Intensive Care Unit (ICU) during the COVID pandemic in the Netherlands, focusing on adaptability and intraPC learning. Transcripts were analyzed using (thematic) template analysis.

**Results:**

Four themes that influenced learning during COVID care were identified: collective uncertainty, social cohesion and a sense of safety, the need for adaptive performance and intraPC learning. During the first wave, collective uncertainty about the unknown disease and the continuation of the crisis urged residents to adapt in order to take care of patients with a disease that was as yet unknown. The combination of collective uncertainty, social cohesion and a sense of safety, and the presence of different disciplines in one department promoted residents’ intraPC learning. However, intraPC learning was not always the matter of course due to the scope of the crisis and the huge numbers of new patients.

**Conclusion:**

Collective uncertainty affected the residents’ adaptability. The combination of collective uncertainty, social cohesion, and the presence of different disciplines in one department promoted the residents’ intraPC learning. An important facilitating factor for both adaptability and intraPC learning is a high level of social cohesion and safety. The physical and psychological proximity of supervisors is an important factor contributing to a safe learning environment. This study provides implications for practice for learning during postgraduate training in non-crisis settings.

**Supplementary Information:**

The online version contains supplementary material available at 10.1186/s12909-022-03868-9.

## Background

When the COVID-19 pandemic created a worldwide public health emergency [1, 2] an enormous influx of extremely ill patients with an unknown disease urged hospitals to suspend plannable care and create new COVID departments. In order to cope with and care for patients with an unknown disease, many doctors from different disciplines had to work together in unknown workflows, with unknown colleagues, and sometimes in new roles. As worldwide knowledge of the coronavirus disease developed only gradually, this required medical professionals to show adaptability and innovative work behavior, for example, in revising existing protocols and creating new ones and in collaborating with doctors from other disciplines. The COVID-19 pandemic thus accelerated the need for doctors to adapt and collaborate in a complex, rapidly changing situation [3].

To adapt to new circumstances, doctors should acquire, integrate, and develop new knowledge and skills in order to solve new problems in their daily work practice while maintaining or improving quality of care [4–7]. This ability is called adaptive expertise or adaptive performance [8, 9]. Professionals with high levels of adaptability demonstrate flexible, innovative, and creative competencies in the domain in which they work [8, 10]. This flexible work behavior helps them adapt to change [11]. The adaptability of professionals is influenced by factors at three different levels: 1) learner/practitioner characteristics, e.g., domain-specific knowledge, skills, regulation processes, and past experiences [9]; 2) task characteristics, e.g., complexity, autonomy, and error-learning [9]; and 3) group/team and organizational characteristics, e.g., support from colleagues, supervisors, and the organization, team learning, and innovation climate [11].

Adaptability alone, however, is not sufficient to guarantee quality of healthcare: it is impossible for doctors to provide comprehensive care as a single professional [12, 13], and particularly during a pandemic, good collaboration among doctors is necessary for them to be able to handle the complexity of care [12, 13]. During the COVID pandemic, doctors from different disciplines were collaborating, which is called *intra*professional collaboration (intraPC) [13]. In the Netherlands, a large proportion of doctors working in COVID departments were residents.

Adaptability and intraPC are not only important during a pandemic, but they are essential competencies for (future) doctors as, with increasing numbers of patients with multimorbidity and rising diagnostic and therapeutic possibilities, the complexity of care is always on the rise [5]. Medical schools, therefore, are trying to find ways to teach their residents adaptability and intraPC skills [9, 12]. See also Table 1.

**Table 1:** Explanation of career paths as graduated doctors

In the Netherlands, medical graduates can continue their careers in medicine in different ways [14]. Most of them apply for a job as ‘doctor not in training’ or apply for a medical residency program. In the position of a ‘doctor not in training’ they work as doctors under supervision of a medical specialist but do not receive education as part of a training program. When admitted to a medical residency program, they become ‘doctors in training’ and they will be trained to become medical specialists. Their primary way of learning is Workplace Learning (WPL) [14, 15].	

During the COVID-19 pandemic, residents and doctors not in training (both referred to as ‘residents’ henceforth) in the Netherlands often worked at COVID or Intensive Care Unit (ICU) departments. The aim of this study is to gain insight into the adaptability and intraPC workplace learning of residents during the COVID-19 pandemic in the Netherlands. The intention here is to learn lessons for the development of adaptive expertise and intraPC learning in postgraduate training in non-crisis settings.

## Method

A qualitative study was carried out by using semi-structured, in-depth interviews from March 2020 up until April 2021. Within this time-span, the COVID-19 pandemic showed three waves in which a large number of patients were admitted to hospital care.

### Context

During the first wave of the pandemic in the Netherlands, plannable care was suspended to deal with the massive influx of COVID patients, with the result that Dutch hospitals consisted chiefly of COVID and ICU -departments at that time. These departments were primarily supervised by medical specialists from disciplines related to COVID care (e.g., internists). These supervisors supervised both residents and medical specialists from disciplines unrelated to COVID care (e.g., gynecologists) that also worked at the COVID and ICU departments. In this article, we will refer to this last group of medical specialists as “guest doctors”.

### Reflexivity and ethical approval

Using an interpretivist research paradigm [16], this study focused on understanding multiple and diverse interpretations of reality. This perspective makes it especially important to pay attention to reflexivity throughout the research process [17], which we did by questioning how our assumptions and perspectives had shaped our data collection, analysis, and interpretation during monthly meetings with all members of the research team. The multidisciplinary research team was valuable during these discussions, as it provided an opportunity to triangulate knowledge and expertise from different professional backgrounds: educational science (CB, CF, WK, MB, MvdH), psychology (RvdG, NL), and medicine (NS, JdG).

The ethical review board of the Dutch Organization of Medical Education (NVMO) approved the study under NERB number 2020.4.4.

### Respondents

Fitting an interpretivist research paradigm [16], we chose a multi-perspective view on the learning of residents [17]. Therefore, we were interested not only in the perspectives of residents, but also in those of supervisors and guest doctors from several disciplines, in order to generate richer data with respect to the adaptability and intraPC learning of residents during COVID care.

Through the Dutch Association of Medical specialists and the Junior Specialists Association, one of the authors (MB), who works as an educational scientist for the Federation Medical Specialists (FMS), obtained an overview of doctors who had given their verbal consent to be approached for research purposes. These doctors worked at a COVID or ICU department in the Netherlands. This list was used for purposive sampling, in order to recruit a diversity of residents and supervisors in different positions and disciplines and working in different hospitals [18]. An information letter and an informed consent form were sent by email to potential respondents by one of the researchers (CB or RvdG). After the first respondents were included, snowballing techniques were used to further diversify our sample of respondents. Data collection was completed when the research group concluded that they had reached meaning saturation to answer the research questions [19].

### Data collection

We conducted semi-structured interviews. A preliminary interview guide, designed by the research group based on literature, was piloted by two researchers (CB and NL) and adjusted afterwards (see Additional file 1 for the final interview guide used). As we were pursuing data saturation of respondents’ perspectives about a similar experience, we decided to focus primarily on respondents’ experiences of the first wave in all interviews. In addition, respondents who provided care in the first and second waves were asked about differences between both waves.

Two researchers (CB and RvdG) performed the interviews as a duo. Due to the COVID measures in place at the time, the interviews were conducted in an online (secured) environment. Each respondent signed the informed consent form prior to the interview. None of the respondents was compensated for their participation. All interviews were audio-recorded and later transcribed. Names and other personal data were not transferred to these transcripts. The interviews were conducted in Dutch and a professional translator translated the interview guide, the quotations and the coding template from Dutch to English at the end of the study.

### Data analysis

Transcripts of the interviews were analyzed using template analysis [20]. This method fitted our research question because it allowed us to combine a solid theoretical foundation with interpretations that were identified from the data. A preliminary template was developed by the research group based on the research question, the main questions from the interview guide and the literature as described in the ‘Background’ of this article. Four codes were determined a priori: adaptability, intraPC learning, individual factors, and context factors. Subsequently, the template was further developed through (re)coding of the interview transcripts and discussion within the research team. Coding was done by CB and RvdG. Each transcript was first coded independently. Next, the coders discussed the transcript to come to a consensus about assigned codes and required adaptations to the coding template. The refined coding template was then used to code another transcript. After each five transcripts were coded, the most recent coding template was used to recode the previous transcripts. The research team was involved throughout this process to discuss changes to the coding template. After all transcripts had been coded, a final template was established by visualizing the interrelationships between the different themes and codes of the template.

## Results

Sixteen interviews were conducted (25–50 minutes per interview) from November 2020 to May 2021 with nine residents, five supervisors, and two guest doctors (Table 2). Respondents worked at nine hospitals all over the Netherlands.

**Table 2:** Description of the respondents

Respondent	Position	Original discipline	Academic (A) vs. non-academic (nA) hospital
1	Resident (not in training)	Emergency	A
2	Resident (not in training)	Internal medicine	A
3	Resident (not in training)	Internal medicine	nA
4	Resident (not in training)	Surgery	nA
5	Resident	Sports medicine	nA
6	Resident	Geriatrics	nA
7	Resident	Internal medicine	nA
8	Resident	Cardiology	nA
9	Resident	Cardiology	nA
10	Supervisor	Internal medicine	A
11	Supervisor	Sports medicine	nA
12	Supervisor	Anesthesiology	nA
13	Supervisor	Internal medicine	nA
14	Supervisor	Geriatrics	nA
15	Guest doctor	Anesthesiology	A
16	Guest doctor	Cardiology	nA

After coding the results, four themes that influenced learning during COVID care were identified: i) collective uncertainty, ii) social cohesion and a sense of safety, iii) the need for adaptive performance and iv) intraPC learning (see Additional file 2 for the final coding template).

### Collective uncertainty

The outbreak of the pandemic was very sudden.

*“That first wave was something that happened to all of us, we had absolutely no accounting for that to happen” (R16, guest doctor).*

Within a very short time span, hospitals had to rearrange their care systems to take care of the “massive influx of patients” (R6, resident) with an unknown disease and with knowledge and treatment developing only slowly. As a result, many changes in schedules, working hours, and locations took place in a short period of time. Residents mentioned that uncertainty about these preconditions was one of the most stressful aspects of their work during the pandemic. “Last minute, other decisions were often made” (R3, resident). Due to the sudden, massive outbreak of an unknown disease, no one knew exactly what to expect and what needed to be done to deal with the crisis. Residents said this was the first time their supervisors and other medical specialists could not answer all their questions.

*“That bit of security, that there is always my supervisor, my back-up, who will know if I don’t know, that dropped away to some extent.”* *(R7, resident).*

This collective uncertainty led to a change in roles and hierarchies. For example a cardiologist (guest-doctor) performing COVID-care as a resident. Learning in another discipline and/or hierarchical role seemed to be very meaningful.

*“It is instructive to see what kind of problems people, young people in this case, can run into when they come to work in a hospital.”* *(R16, guest-doctor).*

Respondents described situations in which a resident “had more COVID knowledge than his/her supervisor” (R8, resident). Related to this were changes in the decision-making process: everyone’s input was taken into account, and as nobody knew the right course of action all ideas were taken into consideration. Respondents also mentioned that, as they were under great pressure to act, they learned to make decisions more quickly than usual. Due to these changes in dynamics, residents were now involved in (management) processes that were usually carried out by medical specialists only.

*“It didn’t matter much anymore whether that literature was put forward by a resident or a staff member [...], but you did take each other seriously because neither of you really knew that much about the matter. It was all discussed very quickly.” (R12, supervisor)*.

Respondents who worked in ICU or COVID departments during both the first and the second wave mentioned that team dynamics changed after the first wave. Collective uncertainty decreased because more knowledge of COVID-19 and its treatment had become available. When plannable care was gradually taken up again and medical specialists returned to care in their own departments, COVID care was run primarily by residents, with some supervisors as a backstop. This caused the level of social cohesion to decrease.

*“During the second wave, things were very different because the regular care had to continue as well. So then we [residents] were essentially responsible for all COVID care in all the COVID departments that were up and running […]. The medical specialists were back to their own wards and their routines. So that’s when it stopped being a collective activity.” (R3, resident).*

### Social cohesion and a sense of safety

Respondents described that they experienced a pleasant working atmosphere and a great sense of team spirit in the COVID and ICU departments during the first wave.

*“I think the team spirit arose naturally in that situation, without having done any explicit team building” (R10, supervisor).*

This was evident in the distribution of tasks and the allocation of patients amongst doctors, which appeared to be taking place in a more organic fashion than in times of non-crisis. A high level of social cohesion was the evident result of working together under pressure with one common goal: providing the best possible care for COVID patients. Furthermore, residents mentioned that they experienced a safe learning climate while providing COVID care.

*“Supervisors communicated well with us, like ‘do clearly state your boundaries, clearly state what you want or don’t want and what you do or do not feel comfortable with’.” (R3, resident).*

Several changes in the way supervision was organized appeared to be related to their experience of safety. First, supervisors were physically more present than in the usual non-crisis situation. Second, supervisors explicitly mentioned that they were available for questions. Third, residents observed that, with supervisors admitting that they also felt insecure about how to deal with COVID patients, they showed themselves to be “vulnerable by delivering care they were no longer comfortable with” (R3, resident). Residents indicated that they felt more comfortable asking any question as no one knew the answer, which was articulated by all doctors. In non-crisis situations, residents feel inhibited by the idea that supervisors will judge their level of knowledge based on the questions they ask. During the pandemic, it made sense that even supervisors had no knowledge of the disease, and residents, therefore, dared to ask anything they wanted to know.

*“So it was just said out loud by everyone: ‘Yes guys, this is a weird situation […], and we [medical specialists] don’t know what’s the matter with all these people either. But we’ve heard about this, so let’s give it a shot.’ That really helped me a lot.” (R7, resident).*

### The need for adaptive performance

As COVID was an unknown disease, there were as yet no guidelines and protocols regarding COVID care. Respondents mentioned that the lack of guidelines and protocols urged them to develop and implement these themselves, involving them, unlike before the crisis, in policy development. Another way in which the respondents’ adaptive performance was stimulated, was by their actively creating an overview to keep the situation manageable.

*“I made guidelines, went through the procedure with the nurses, made a notice board for the hallway with the important phone numbers and who does what where, and listing the medication that we still gave at the time.” (R6, resident).*

Residents attributed their ability to adapt to the new situation to various aspects: previous work experience, clinical reasoning skills, personality, and the social cohesion that prevailed during the pandemic. Being able to adapt allowed them to deal with the uncertainties that came with the pandemic. It was valuable for them to realize that they could manage their work during a crisis situation, which boosted their self-confidence and benefited their professional development.

*“So many things I was dealing with. At first, it was just like ‘Wow, I’m really doing this!’”. (R6, resident).*

COVID care specifically accelerated the need for developing new knowledge and skills, thus developing a flexible attitude from all doctors. Supervisors explained that residents need a flexible attitude even in non-crisis situations because they often encounter new situations.

*“I think that, because of their age, residents aren’t that stuck in their ways yet, and so they find it a lot easier to step out of their comfort zone. In a sense, they’re always out of their comfort zone, as they’re still learning. They’re new to the hospital, and so they’re used to dealing with new things all the time.” (R13, supervisor).*

Some residents indicated that they had explicitly learned to set their own boundaries and that their career choice had been confirmed by working as a doctor at a COVID or ICU department during the crisis. They also noted that once they had become familiar with the disease and its methods and protocols, “the work itself was relatively easy” (R8, resident), and routines developed quickly. Some residents, therefore, worried that working in COVID care for a long period of time would limit their possibilities for learning.

*“I am a bit afraid that I didn’t acquire as much medical knowledge as I should have at that point in my training program […]. I saw so many [COVID patients], your learning curve does end at some point. I just missed a lot of training moments for dealing with other internal patients.” (R8, resident).*

### IntraPC learning

The majority of participants mentioned that the presence of doctors from different disciplines in one department was beneficial for intraPC because this made it easy for them to consult someone with specific expertise.

*“Normally you would ask the ENT-doctor for a consultation, and he would have to come by, but he was already in the department, so he could help out.”* *(R2, resident).*

Residents appeared to ask mostly medical questions to their supervisors during (formal) supervision moments; they asked the easier or more practical questions to other residents. In addition, residents indicated that they also collaborated with residents from other disciplines and learned from them by having conversations about their background, expertise, experience, and ideas, which mainly took place during the quieter moments.

*“Because of the different backgrounds, we purposefully asked questions to certain people. And so, as I had worked in an emergency care department for a year, the internal medicine resident approached me sometimes, saying ‘How do you think I should handle this in the emergency room?’ And I said ‘Well, this way and that’. So your background and previous experience were deliberately used.” (R1, resident).*

The interviews revealed that intraPC between residents mainly occurred in specific situations. One internist in-training, for example, explained how she consulted a gynecologist in-training “I consulted a gynecologist in-training who was working at the same COVID-department when I encountered a pregnant COVID patient” (R2, resident). However due to lack of time and protective equipment, doctors did not (or no longer) visit patients together. In this regard, some mentioned that guest doctors were more inclined to simply refer their patient to a doctor from an appropriate discipline rather than consult that doctor to broaden their intraprofessional knowledge and ability by taking care of that patient themselves.

Similarly, respondents indicated that guest doctors at the ICU and COVID departments only called upon residents on rare occasions and that the questions they asked were mostly about practicalities, such as “where do I report this in the electronic patient file?”. In this sense, there appeared to be limited reciprocity regarding the exchange of domain-specific knowledge between residents and medical specialists.

*“I asked the anesthetists [supervisors] like ‘Well, would you show me how to use an ultrasound when placing an IV. I would like to learn that’. And the other way around it was more like ‘Well, you do it because I don’t know how to’. But not like ‘Diabetes, that’s interesting, can you tell me a little more about it?’ No, there wasn’t really any curiosity like that.” (R7, resident).*

Some characteristics of the COVID situation appeared to impede intraPC learning: the high pressure and pace of COVID care, the reduced opportunities for providing care together and the suspension of joint education sessions. This last characteristic was related to the limited availability of protective equipment and to COVID-related constraints such as the limited presence of doctors on the ward. Some respondents mentioned that they were assigned to patients and got so involved with these patients that they hardly ever spoke to colleagues; they “just did their job” (R16, guest-doctor). These respondents explained that they looked up the necessary information themselves or consulted their own network outside the hospital.

“*It was something you did, as you didn’t interact much with the others. We didn’t meet anymore at all. […] You had to do it yourself as a doctor,* to *find all the information you needed. There just weren’t any other moments.” (R16, guest-doctor).*

### Post crisis intraPC

Working in the same department during the COVID crisis appeared to have had a reinforcing effect on post-crisis collaboration. Respondents mentioned that they communicated more easily and openly with colleagues from other disciplines they had met during COVID care, even though they had returned to their own wards.

*“The neurosurgery people, for example, I didn’t know those people at all because you never meet them normally. But at the COVID department I had worked with this guy for four weeks. And now when I call the department and I happen to speak to him on the phone, I just say “Hey [name], how are you?” That makes it so much easier to work together.” (R2, resident).*

## Discussion

This study aimed to gain insight into the adaptive performance and intraPC learning of residents during the COVID-19 pandemic. The first wave of the pandemic was characterized by a collective uncertainty among all doctors involved and a high level of social cohesion and a sense of safety on COVID and ICU wards. The collective uncertainty forced supervisors and residents to adapt as they had to find solutions and create an overview within an unpredictable crisis situation. The experience of being able to adapt to uncertain, changing circumstances appeared to increase the residents’ self-confidence. The combination of collective uncertainty, a high level of social cohesion and a sense of safety, and the presence of doctors from different disciplines within COVID departments also promoted residents’ intraPC learning. Though this was not always the matter of course: due to the scope of the crisis and the huge numbers of new patients, it was sometimes difficult to collaborate with other doctors and learn from them.

This study showed that the urgency of caring for extremely ill patients with an unknown disease created collective uncertainty and prompted supervisors and residents to adapt. This is in line with prior studies which have shown that adaptability is characterized by coping with stressful situations or emergencies and dealing with uncertainty and changing circumstances [21, 22]. In the first wave, residents working in COVID or ICU departments faced stressful, uncertain circumstances and provided care to large numbers of COVID patients within a limited time span. This turned out to be conducive to their learning process. The residents’ adaptive expertise appeared to be particularly stimulated by their growing domain-specific knowledge of COVID-19, the task complexity involved in COVID care, and their working with supportive colleagues who stimulated team learning. This is in line with earlier studies into adaptive expertise and adaptive performance which concluded that the extent of knowledge and task complexity is important for (developing) adaptive expertise [9] and that team learning is an antecedent for adaptive performance [11].

In the subsequent waves, more knowledge of how to manage the disease had become available, and working practices had been laid down into protocols. After the first wave, therefore, doctors worked in COVID care with increasing efficiency, turning COVID care into a routine task. Working towards mastering COVID care by performing all necessary actions to the best of their ability and becoming “routine experts in covid care” appears to be beneficial for residents in the short term because this pushes them to perform with the greatest efficiency and effectiveness. In the longer term, however, when the innovation dimension was excluded or undervalued, opportunities for developing adaptive expertise reduced [9]. In addition, our results showed that, after the first wave, the large flow of patients and especially the performance of what had now become routine tasks appears to have led to a decreased motivation to work in the COVID department and no longer appealed to the adaptability. This supports previous research which claims that adapting to new situations, adaptability, can be stimulated by presenting information in multiple contexts [23].

The collective uncertainty among first-wave doctors not only promoted adaptability but also contributed to intraPC learning. Previous research has shown that there are many barriers to intraPC learning, such as a high level of hierarchy in the workplace, lack of awareness of intraPC learning opportunities, and unidirectional learning [24, 25]. The presence of different disciplines in one location, therefore, does not necessarily result in intraPC learning [24]. Our research showed, however, that the presence of different disciplines in one COVID /ICU department led to lower thresholds for collaboration and encouraged residents to consult intraprofessional colleagues, both during and after the first wave.

We found two possible explanations for this. One possible explanation is that the combination of collective uncertainty, psychological proximity, and an extraordinary degree of social cohesion during work in the same department in a pandemic crisis stimulates cross-boundary teaming [26]. Our study shows that this creates a strong team spirit, which positively influences interpersonal relationships. IntraPC turned out to have improved after the pandemic, with respondents reporting that their thresholds for initiating interactions with intraprofessional colleagues, with whom they had worked in the same COVID /ICU department, decreased once they had returned to their own workplaces. This could foster future intraPC learning.

Another possible explanation could be the occurrence of constructive power dynamics in COVID departments. Power dynamics describe “the way in which power impacts the interaction of two or more people or groups” [25, 27] and can either have a constructive or nonconstructive manifestation and, consequently, a corrosive or conducive effect on intraPC learning [25]. Our study shows that different constructive power dynamics were at work in COVID/ICU departments, such as everyone’s shared lack of knowledge of COVID-19, the distribution of roles and responsibilities based on equity without any inter-discipline supremacy, sincere and equal collaboration, and everyone’s accessibility for consultation. Positive power dynamics are a major contributor to a culture of sincere equal intraPC. However, our research also showed that intraPC learning could be limited by the high workload and various practical limitations.

Most COVID /ICU departments in the first wave were considered a safe psychological working and learning environment, which promoted both the adaptability and the intraPC learning of residents. Previous research already showed that a supportive learning climate affects learners’ motivation, self-confidence, and overall moral and academic achievements [28–30]. Our study shows that the perceived psychological safety was facilitated by the proximity of supervisors in two ways: physical proximity and, more importantly, psychological proximity. Physical proximity occurred because most supervisors were available on-site rather than on call, and psychological proximity occurred because supervisors repeatedly instructed residents to approach them with questions and were explicitly transparent about their clinical uncertainty regarding COVID patient cases.

Such psychological proximity bridges the hierarchical gap between residents and supervisors and influences the residents’ perception of clinical uncertainty. Although recognizing and coping with clinical uncertainty is part of the doctors’ job, being able to accept and deal with uncertainty is something many find challenging [31]. Mutual trust and psychological proximity can make it easier for residents to stretch themselves beyond their comfort zone. The pandemic “forced” supervisors to show themselves to be vulnerable by admitting that they were uncertain as well and did not have all the answers. Residents appreciated this vulnerability, as it confirmed to them that it was okay to feel uncertain and to ask questions. Prior research confirms that supervisors’ willingness to engage collegially with residents and disclose their vulnerabilities leads to enhanced mutual trust, which fosters learning [32]. As most postgraduate training programs consist of short rotations, in which opportunities for developing supervisor-trainee trust relations are scarce, it is recommended to explore ways to foster a culture of trust [32]. Our study provides a valuable complement by providing implications for practice, based on learning during COVID, for learning during postgraduate training in non-crisis settings.

### Implications for practice

In facilitating the enhancement of adaptability and intraPC learning during postgraduate training, the following ideas might be helpful.

First, create a safe learning environment by investing in social cohesion and team spirit, being easily approachable to other disciplines, and responding respectfully to questions. Show the human factor and stimulate the dialogue. For example, by having sincere attention and proactively asking for personal opinions while discussing a patient case.

Second, create a culture in which everyone can express themselves freely and in which supervisors can express clinical uncertainty, for example, by being transparent, open, vulnerable, and honest. For instance, by having supervisors sharing their own uncertainties during patient care and stimulating others to do the same.

Third, deliberately apply two modes of supervisor proximity: physical proximity and psychological proximity. If possible, be physically close, but at least be accessible to residents as a supervisor. Supervisors can, for example, mention explicitly that they are available for questions. Listen to residents’ questions and also encourage them to find their solutions, perhaps with their intraprofessional colleagues. A resident we spoke to as part of the pilot interviews suggested that residents can organize their own multidisciplinary team discussion with a supervisor sitting in the background, only giving their own input at the end.

Fourth, proactively learn from different perspectives by working at another specialty department or in another hierarchical role. For example, having supervisors putting themselves in the shoes of a resident, possibly even from another discipline. And experience and learn from working in a different role. One cardiologist who worked as a resident during the crisis, recommended all supervisors to experience working as a resident every once in a while.

Fifth, learn from uncertainty. Train your flexibility and adaptability, by doing new things, by simulating situations with many uncertainties in which supervisors and residents learn together in situations where protocols and guidelines could not be applied, or by participating in parts of the care process with which you are unfamiliar. For example by simulating a crisis situation whereby residents and supervisors learn together in unknown situations, with unknown workflows and protocols and guidelines.

### Strengths and limitations

A strength of this study is its three types of triangulation: a) data source triangulation: triangulation in perspectives on the residents’ learning was established by interviewing residents, supervisors, and guest doctors; b) investigator triangulation: all interviews and the coding process were performed by two researchers, thus combining two perspectives to generate a thorough analysis; and c) research group triangulation: our research was conducted in a multidisciplinary team, with the different professional knowledge domains and backgrounds operating as a form of triangulation [17].

A limitation of our research might be the time gap between when care was provided during the pandemic and when the interviews were held: some respondents were interviewed in December 2020, while the first COVID wave started in March and ended in May 2020. This may have resulted in recall bias and incomplete respondents’ stories. The scale of the pandemic, however, made it impossible to conduct interviews earlier on. Another limitation of our research might be potential inclusion bias, due to, for example, people who do have strong opinions about learning and collaboration in general, or specifically during this crisis. We mitigated this limitation by recruiting a diversity of residents and supervisors in different positions and disciplines and working in different hospitals. Further, the interviews were conducted in Dutch and a professional translator translated the interview guide, the quotations and the coding template from Dutch to English. Although there can always be some differences in word usage in translations, we believe the quotations accurately reflect the interviews and do justice to what respondents said. Therefore we think we have minimalized the chance to lose information.

## Future research

Further research is needed to determine whether and how the implications for practice will help to improve adaptability and intraprofessional collaboration during non-crisis situations.

## Conclusions

Collective uncertainty affects the adaptability of residents. The lack of guidelines and protocols ensured doctors developed and implemented these themselves, which accelerated the need for developing a flexible attitude and being adaptable. The combination of collective uncertainty, social cohesion, and the presence of different disciplines in one department can promote residents’ intraPC learning. An important facilitating factor for both adaptability and intraPC learning is a high level of social cohesion and safety, as this was experienced during the first COVID wave. The physical and psychological proximity of supervisors is an important factor contributing to a safe learning environment. This study provides implications for practice for learning during postgraduate training in non-crisis settings.

## Supplementary Information

**Additional file 1.**

**Additional file 2.**

## Data Availability

All data generated or analyzed during this study are included in this published article. The data that support the findings of this study are available from the corresponding author upon reasonable request.

## Abbreviations

IntraPC: Intraprofessional Collaboration
ICU: Intensive Care Unit
WPL: Workplace Learning

## References

[1] Zangrillo A (2020). Fast reshaping of intensive care unit facilities in a large metropolitan hospital in Milan, Italy: facing the COVID-19 pandemic emergency. Crit Care Resusc.

[2] Organization WH (2020). Novel coronavirus (2019-nCoV) situation reports.

[3] Natale JE (2020). Interprofessional/interdisciplinary teamwork during the early COVID-19 pandemic: experience from a children's hospital within an academic health center. J Interprof Care.

[4] Eva KW (2005). What every teacher needs to know about clinical reasoning. Med Educ.

[5] Mylopoulos M, Regehr G (2009). How student models of expertise and innovation impact the development of adaptive expertise in medicine. Med Educ.

[6] Norman G (2005). Research in clinical reasoning: past history and current trends. Med Educ.

[7] Cutrer WB (2017). Fostering the development of master adaptive learners: a conceptual model to guide skill Acquisition in Medical Education. Acad Med.

[8] Hatano G, Inagaki K, Stevenson H, Azuma H, Hakuta K (1986). Two courses of expertise in child development and education in Japan.

[9] Bohle Carbonell KB (2014). How experts deal with novel situations: a review of adaptive expertise. Educ Res Rev.

[10] Bransford JD, Brown AL, Cocking RR (2000). How People Learn: Brain, Mind, Experience, and School.

[11] Park S, Park S (2019). Employee adaptive performance and its antecedents: review and synthesis. Hum Resour Dev Rev.

[12] Hall P, Weaver L (2001). Interdisciplinary education and teamwork: a long and winding road. Med Educ.

[13] Gilbert JH, Yan J, Hoffman SJ (2010). A WHO report: framework for action on interprofessional education and collaborative practice. J Allied Health.

[14] Ten Cate O (2007). Medical education in the Netherlands. Med Teach.

[15] Tynjala P (2008). Perspectives into learning at the workplace. Educ Res Rev.

[16] Bunniss S, Kelly DR (2010). Research paradigms in medical education research. Med Educ.

[17] Barry CA (1999). Using reflexivity to optimize teamwork in qualitative research. Qual Health Res.

[18] Coyne IT (1997). Sampling in qualitative research. Purposeful and theoretical sampling; merging or clear boundaries?. J Adv Nurs.

[19] Hennink MM, Kaiser BN, Marconi VC (2017). Code saturation versus meaning saturation: how many interviews are enough?. Qual Health Res.

[20] Tabari S, King N, Egan D (2020). Potential application of template analysis in qualitative hospitality management research. Hosp Soc.

[21] Oprins EAPB, van den Bosch K, Venrooij W (2018). Measuring adaptability demands of jobs and the adaptability of military and civilians. Mil Psychol.

[22] Pulakos ED (2002). Predicting adaptive performance: further tests of a model of adaptability. Hum Perform.

[23] Bransford JD, Schwartz DL (1999). Chapter 3: rethinking transfer: a simple proposal with multiple implications. Rev Res Educ.

[24] Looman N (2020). Chances for learning intraprofessional collaboration between residents in hospitals. Med Educ.

[25] Looman N (2022). Exploring power dynamics and their impact on intraprofessional learning. Med Educ.

[26] Edmondson AC, Harvey JF (2018). Cross-boundary teaming for innovation: integrating research on teams and knowledge in organizations. Hum Resour Manage Rev.

[27] Simons J (2013). Power, resistance, and freedom. A companion to Foucault.

[28] Abraham R (2008). Students' perceptions of learning environment in an Indian medical school. BMC Med Educ.

[29] Saito A (2010). Learning climate in dental hygiene education: a longitudinal case study of a Japanese and Canadian programme. Int J Dent Hyg.

[30] Lucas CA, Benedek D, Pangaro L (1993). Learning climate and students achievement in a medicine clerkship. Acad Med.

[31] Simpkin AL, Schwartzstein RM (2016). Tolerating uncertainty - the next medical revolution?. N Engl J Med.

[32] Castanelli DJ (2022). How trainees come to trust supervisors in workplace-based assessment: a grounded theory study. Acad Med.

