# Supplementary material for: Adaptability and learning Intraprofessional collaboration of residents during the COVID-19 pandemic
Source: BMC Med Educ. 2022 Nov 12;22:782. doi: 10.1186/s12909-022-03868-9 (PMC9652594; doi:10.1186/s12909-022-03868-9)
Supplement: Supplementary file 1 — Additional file 1. [file 12909_2022_3868_MOESM1_ESM.docx]

**Additional file 1**

Interview Guides

**Interview Guide for Residents**

| **Categories** | **Primary questions** | **(Possible) supplementary questions** |
| --- | --- | --- |
| **Introduction** | Would you introduce yourself?   - Which residency program are you taking? - What is your work experience?   Can you tell us about your activities at the COVID/ICU department from March 2020 onwards? | - What else did you experience?   *Alternatives*   - You mentioned […], could you clarify that? - Can you give an example of that? |
| **Individual development and adaptability** | How did your activities in the COVID/ICU department influence your professional development?  Were there things that you needed to do differently than in the normal situation? If so, what was different?   - How did you deal with these differences? - Did you come up with new activities or solutions yourself?   Who or what was helpful when performing things differently?  Who or what was hindering when performing things differently?  Were there any times when you were surprised by your own activities?  What were the supervision arrangements?  In sum: what was helpful for developing expertise during the first wave? And what was hindering? | - What kind of knowledge/skills/abilities were required from residents? - Did you have any previous experience with radical change or unpredictable situation? If so, did these experiences help you in the current situation? - Did you come up with things yourself, or were you not allowed to do so?   *Alternatives:*   - You mentioned […], could you clarify that? - Can you give an example of that? - What was enabling? - What was hindering? - Do I understand correctly that …? - It is important to you that …? |
| **IntraPC learning** | Can you tell us about collaboration with colleagues during COVID care?  What did you like or dislike about this?  Did you experience any differences in collaborating with different colleagues?   - Differences between disciplines? - Differences between residents/ supervisors/guest doctors? - Differences with the normal situation?   What did you learn from collaborating with colleagues from different disciplines?   - Did colleagues challenge you to perform differently and/or learn new things? - Did you challenge colleagues to perform differently and/or learn new things?   Who or what was helpful during collaboration with colleagues?  Who or what was hindering during collaboration with colleagues? | - To what extent do you recognize these intraPC activities from the normal situation? - Did the crisis influence the degree of connection with colleagues? - Did the crisis impact your outlook on learning and/or collaboration?   *Alternatives*   - Do you have similar experiences in your collaboration with other disciplines? - You mentioned […], can you clarify that? - Do you have an example of that? - What was your contribution? - Why do you think that is important? |
| **First vs. second wave** *This category was added in a final version of the interview guide* | In previous interviews, we heard that there were differences between the first and second waves. How do you experience this?   - How do you explain these differences? | - What is the biggest difference according to you? - Do you have an example of this? |
| **Future** | What lessons could be learned from your experiences during COVID care?  How could other residents acquire the same knowledge/skills/abilities when working in regular care? | *Alternatives*   - You mentioned […], can you clarify that? - Can you give an example of this? - How do you envision this? - Did I understand correctly that …? |
| **To conclude** | Is there anything that you would like to add or change in response to everything we discussed? |  |

**Interview Guide for Supervisors and Guest Doctors**

| **Categories** | **Primary questions** | **(Possible) supplementary questions** |
| --- | --- | --- |
| **Introduction** | Could you introduce yourself?   - What is your medical specialism? - What is your experience with supervising residents?   What were your tasks and responsibilities with regard to guiding residents? To what extent did this differ from your normal tasks?  What were the tasks and responsibilities of residents during COVID care? How did this differ from the regular situation? | *Alternatives*   - You mentioned […], could you clarify that? - Can you give an example of that? |
| **Individual development and adaptability of residents** | What did you see residents do during the first wave?  What was different in this compared to before?  Did you see any differences between residents during the first wave? What differences?  How did the residents’ practices differ from one another?  In what ways did residents deal with this new way of working? What do you think this depended on?  To what extent did you play a role for the residents in your department during the first wave? What role?  Was there a difference between residents from your own discipline and residents from other disciplines?   - What appeal did these residents in your department make on you (and your fellow supervisors)?   Have you learned anything from residents? What? How did you experience this?  Do you think some competencies received more attention during the first wave than before? In case of change, how did this come about?  What did this mean for the residents’ development and supervision?  Are there any competencies that did not get much attention during the first wave and that need to be given more attention now?  Have you wondered about something residents did during the first wave? What did they do? How did you experience this? | - Why did this happen in this way? - What kind of knowledge/skills/abilities were required from residents? - How did residents deal with these new experiences/practices? - What helped residents?   *Alternatives:*   - You said […], could you explain that further? - Could you give an example? - What is conducive in this? - What is hindering it?   *Concluding question:*   - Do I understand correctly that… - It is important for you that…? |
| **IntraPC learning of residents** | How did residents collaborate with other disciplines at your department during the first wave?  To what extent did you play a role in collaboration among residents or with other physicians in your department during the first wave? What role?  Was there a difference in this between residents from your own discipline and residents from other disciplines?  How was the collaboration between you and the residents you supervised during first wave? To what extent was it different from before?  What do you think helped residents in collaborating with other physicians during the first wave? Who or what was supportive in this?  What do you think was difficult or got in the way for residents in collaborating with other physicians during the first wave? Who or what could have helped them?  Did you also learn things from residents in terms of collaborating with physicians from other disciplines during the first wave? If so, what? (How did you experience this?) | - Could you tell me more about that? - To what extent do you recognize this during IPC in non-crisis time? - During the crisis, did you experience a difference between collaboration of doctors from different disciplines? What difference? - To what extend did the environment (colleagues, supervisors, physical location, etc.) contribute to the professional development of residents? - Did this differ during crisis than before crisis times? If so, in what way?   *Alternatives:*   - You said […], could you explain that further? - Could you give an example of that? - What was your role? - Why do you think that is important?   *Concluding question:*   - Do I understand correctly that… - It is important for you that…? |
| **First vs. second wave** *This category was added in a final version of the interview guide* | In previous interviews we heard that there were differences between the first and second waves. How do you experience this?  How do you explain these differences? | - What is the biggest difference according to you? - Do you have an example of this? |
| **Future** | What lessons could be learned from your experience as a supervisor during COVID care?  How could other residents acquire the same knowledge/skills/abilities when working in regular care? | *Alternatives*   - You mentioned […], can you clarify that? - Can you give an example of this? - How do you envision this? - Did I understand correctly that …? |
| **To conclude** | Is there anything that you would like to add or change in response to everything we discussed? |  |
