# Supplementary material for: Adaptability and learning Intraprofessional collaboration of residents during the COVID-19 pandemic
Source: BMC Med Educ. 2022 Nov 12;22:782. doi: 10.1186/s12909-022-03868-9 (PMC9652594; doi:10.1186/s12909-022-03868-9)
Supplement: Supplementary file 2 — Additional file 2. [file 12909_2022_3868_MOESM2_ESM.docx]

**Additional file 2**

*
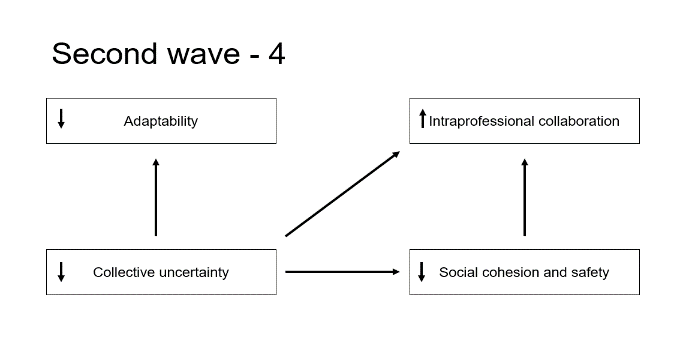
*
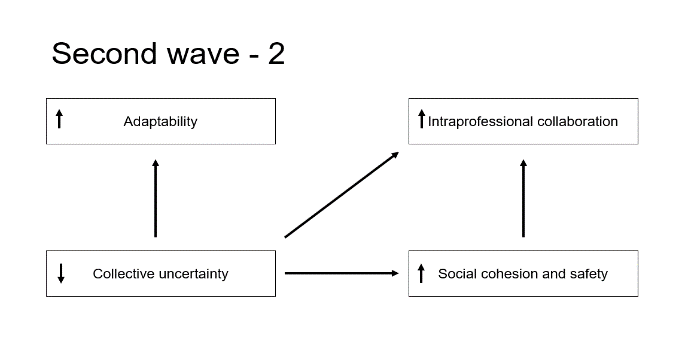

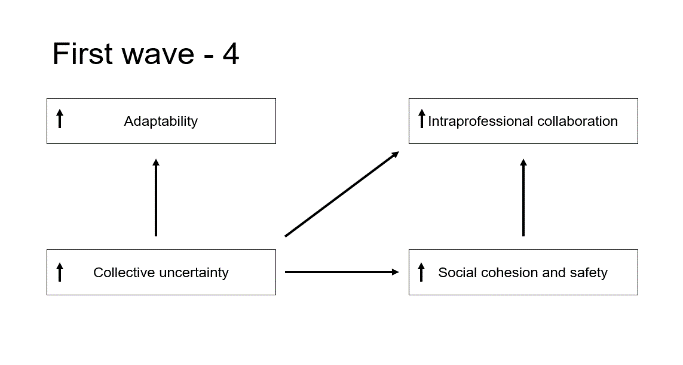

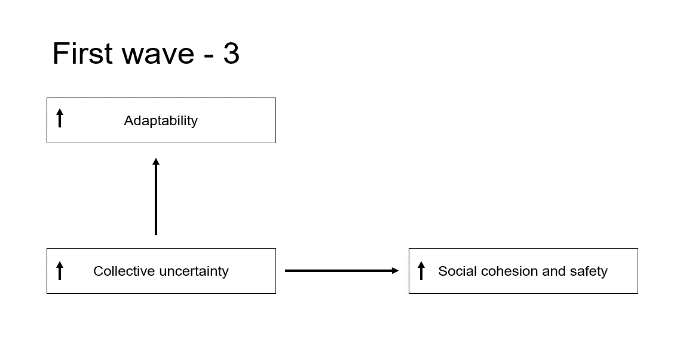

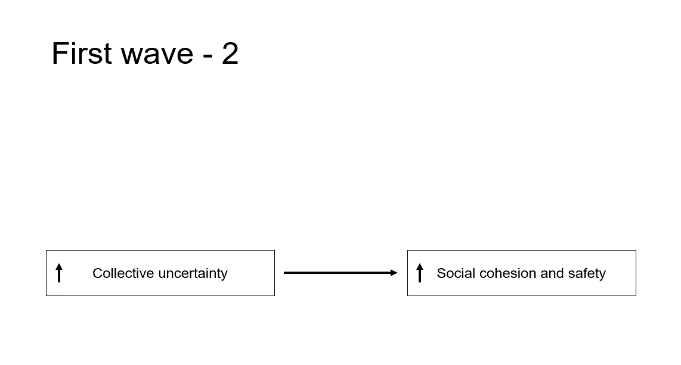

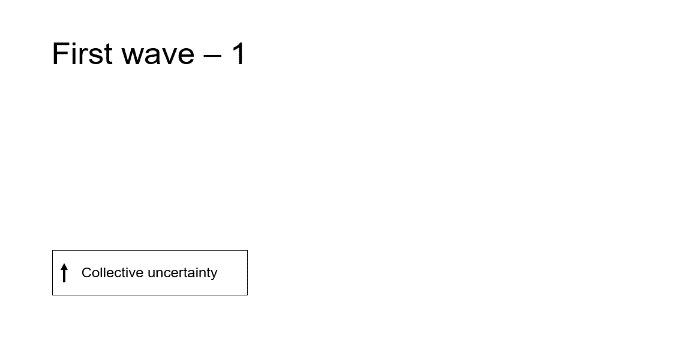
Coding Template

*
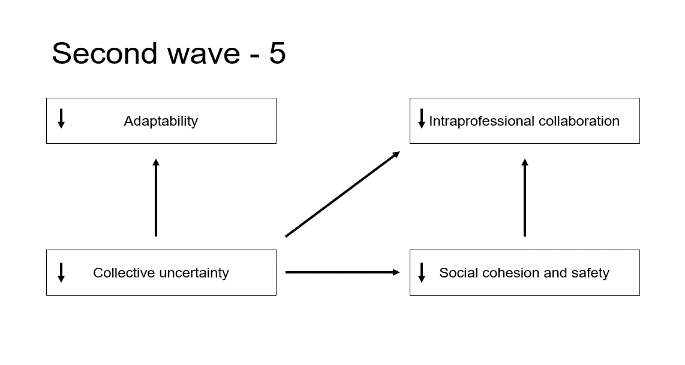

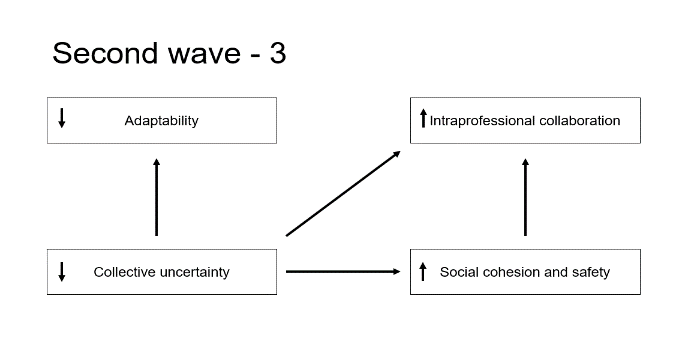

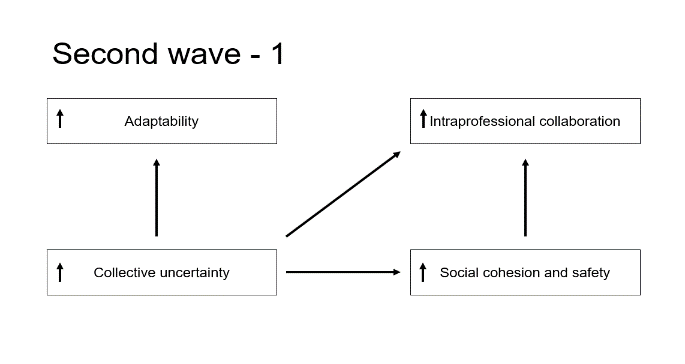
*

*Figure 1.* Schematic representation of the coding template

Above, one can see a schematic representation of the coding template. This representation shows the main concepts of this study and its interrelationship and development during the COVID crisis. An explanation for each phase follows below, distinguishing between the first and second waves.

First wave 1: the first Covid-wave created collective uncertainty among doctors: no one knew exactly what to expect and what needed to be done to deal with the crisis.

First wave 2: collective uncertainty created social cohesion among doctors by working together under pressure with one common goal: providing the best possible care for COVID patients. As no one did know the right course of action, everyone’s input was considered valuable. This created a sense of safety.

First wave 3: as COVID was an unknown disease, there were no guidelines and protocols regarding COVID-care. Doctors had to develop and implement these themselves. Thus, COVID-care accelerated the need for all doctors to develop a flexible attitude and be adaptable.

First wave 4: this resulted in the presence of doctors from different disciplines in COVID-/ ICU-departments. This seemed beneficial for intraPC because this presence made it easy for doctors to consult someone with specific expertise.

Second wave 1: When the second wave arrived, at first, the situation seemed quite the same as at the end of wave 1.

Second wave 2: However, this situation soon changed. Collective uncertainty decreased since more knowledge of COVID-19 and its treatment had become available.

Second wave 3: doctors became familiar with the disease and its methods and protocols and routines developed quickly. As a result, adaptability was no longer called upon.

Second wave 4: when plannable care was picked up again, medical specialists returned to their own departments leaving COVID care, which was now run primarily by residents. This caused the level of social cohesion to decrease.

Second wave 5: Since COVID-care was now primarily performed by residents, there was less intraPC at COVID-/ICU-departments.

**Table 1**

*Elaboration of the code tree from our research*

| **Theme** | **Code** |
| --- | --- |
| Collective uncertainty | Crisis   - Crisis management   Social Debate  Changes   - Decision-making   - Speed of decision making   - Every voice counts - Roles and hierarchy - Way of working   - Reorganizing departments   - Supervision set-up   - Induction process   - Scheduling and planning   - Team composition |
| Social cohesion and a sense of safety | Team spirit  Security  Humor  Being vulnerable  Safe environment  Appreciation   - Society - Organization - Work environment/colleagues   Supervision   - Proximity - Expectations   - Based on year of education   - Based on discipline |
| The need for adaptive performance | Working in a protocol-based manner  Going with the flow  Clinical reasoning  Dealing with uncertainty  Coming up with solutions  Creating an overview  Individual characteristics   - Personality - Work experience - Self-confidence - Feeling competent - Willingness - Coping - Challenge - Stress/emotions   - Survival mode   - Fatigue |
| IntraPC learning | How does one collaborate IP?   - Making agreements about (organizational) tasks - Meeting each other - Seeing patients together - Allocating patients   Expectations during IPC learning based on   - Job level - Discipline   Who learns what from whom?   - Medical knowledge and skills - Logistics |
